# Construction and application of coordination ability evaluation tool for children aged 4–5.9 years old

**DOI:** 10.3389/fpubh.2026.1698428

**Published:** 2026-02-11

**Authors:** Weifu Chen, Na Li

**Affiliations:** 1 Department of Physical Education, Beijing Union University, Beijing, China; 2 School of Physical Education, Shandong University of Science and Technology, Qingdao, China

**Keywords:** assessment criteria, basic motor skill, coordination ability, evaluation index system, preschool children

## Abstract

**Background:**

Coordination ability is a critical component influencing children's basic motor skills and participation in physical activities. Positive experiences formed during early movement practice are essential for lifelong health.

**Purpose:**

This study aimed to scientifically construct a systematic and user-friendly evaluation tool for assessing coordination ability in children aged 4.0–5.9 years, thereby enhancing understanding and promoting targeted development in family and kindergarten activities.

**Methods:**

The evaluation indicators were determined through literature review, structured expert interviews, expert interviews, and the Delphi method. Sixteen cities in Shandong Province were sampled using a stratified random cluster sampling approach. A total of 1,068 valid samples were obtained (530 children aged 4.0–4.9 years and 538 children aged 5.0–5.9 years). Statistical analyses were performed using SPSS 26.0 and AMOS 26.0, including exploratory and confirmatory factor analyses to test model validity.

**Results:**

The structural model of coordination ability demonstrated good fit indices. Coordination ability was found to consist of six key dimensions: balance, spatial orientation, rhythm, perceptual judgment, limb coordination, and limb movement range. Evaluation criteria for both 4- and 5-year-old groups were established. Girls exhibited higher overall coordination scores than boys. Boys in the 4-year-old group showed superior perceptual and judgment ability, while boys in the 5-year-old group performed better in perceptual judgment and spatial orientation.

**Conclusion:**

(1) The constructed evaluation tool accurately reflects children's coordination development. (2) The established single and composite scoring criteria effectively distinguish coordination levels among children aged 4–5.9 years. (3) Gender differences exist, with girls generally outperforming boys, though developmental asynchrony is evident across coordination components.

## 1 Introduction

Early childhood (4–5.9 years old) is a critical period for the development of basic motor skills, and coordination ability, as the core foundation of these skills, directly affects children's participation in daily physical activities, cognitive development, and long-term health outcomes (1, 2). With the global emphasis on early childhood education quality-evidenced by China's 20th National Congress of the Communist Party of China highlighting the strategic importance of strengthening early childhood education (3) -the assessment and promotion of children's coordination ability have become key focuses in preschool education and sports science.

Notably, coordination ability in children aged 4–5.9 years presents distinct developmental traits, manifestations, and influencing factors that underpin the need for targeted research.

(1) Characteristics: this stage is a “sensitive period” for coordination development-children's coordination improves rapidly with physiological maturation (e.g., enhanced neural control of limbs and vestibular organ stability) but shows obvious age and gender asynchrony. For 4-year-olds, static balance (e.g., single-leg stance) and limb flexibility develop faster, while 5-year-olds exhibit accelerated progress in dynamic balance (4). Gender differences also emerge: girls outperform boys in limb flexibility and static balance, while boys show advantages in reaction speed and spatial tasks.
(2) Forms of manifestation: coordination is reflected in daily motor behaviors integrated with six core dimensions. Specifically, it includes balanced movements (e.g., tiptoe straight-line walking, single-leg stance), rhythmic responses, spatial operations (e.g., curve walking), perceptual reactions (e.g., hand reaction), limb synergy (e.g., fist-kick movements), and limb extension (e.g., single-leg seated forward bend). These manifestations align with kindergarten daily activities, ensuring practical relevance.
(3) Influencing factors: it is shaped by innate and acquired factors. Innate factors include genetic physiological maturation (e.g., muscle-ligament elasticity affecting limb movement range, vestibular development supporting spatial orientation) (5). Acquired factors involve family environment (e.g., parental guidance on physical activities), kindergarten curriculum (e.g., frequency of rhythm games), and motor experience (e.g., girls' dance participation enhancing flexibility, boys' ball games improving directional kicking) (6, 7).

Existing research on children's coordination ability has made progress in multiple dimensions. Quantitative studies have used tools such as the Test of Gross Motor Development (TGMD) series to assess coordination levels across age groups (8), while qualitative research has observed coordination performance in naturalistic settings like kindergarten activities (9). Regional studies have compared coordination development between urban and rural children (10), and longitudinal tracking has explored age-related changes in coordination components (11). However, three critical gaps remain in the specialized literature: first, lack of age-specific and culturally adapted assessment tools. Most existing tools (e.g., TGMD-2) were developed based on Western populations and may not fully align with the motor development norms of Chinese children aged 4–5.9 years (12). For example, Western tools often include complex ball-playing tasks that are less common in Chinese preschool curricula, leading to low feasibility in local testing (13). Second, incomplete structural definition of coordination ability for preschoolers. Previous studies have fragmented coordination into single components (e.g., balance or rhythm) but failed to systematically integrate multiple dimensions (e.g., spatial orientation, perceptual judgment) into a unified evaluation framework (14). This fragmentation hinders a comprehensive understanding of how coordination components interact to support overall motor development. Third, insufficient theoretical grounding in assessment tool construction. Most tools rely on empirical testing without integrating core theories such as Piaget's schema coordination theory (1952) or the Complex Adaptive Systems (CAS) theory, which explain how children's nervous systems, sensory organs, and environmental interactions shape coordination (15). This lack of theoretical support reduces the tool's explanatory power for coordination development mechanisms.

Against this backdrop, this study aims to address these gaps by: (1) constructing a systematic coordination ability evaluation index system for Chinese children aged 4–5.9 years, integrating six core components (balance ability, spatial orientation ability, rhythm ability, perceptual judgment ability, limb coordination ability, and limb movement range) based on CAS theory and Piaget's schema theory; (2) developing age-specific (4- and 5-year-old groups) and easy-to-implement assessment standards, tailored to the daily activity contexts of Chinese kindergartens; (3) verifying the tool's validity and reliability through large-sample testing in Shandong Province, to provide a practical and theoretically grounded instrument for preschool educators and researchers. The findings are expected to fill the literature gap in culturally adapted, theory-driven coordination assessment for young Chinese children and support targeted interventions to promote their basic motor skill development.

## 2 Methods

### 2.1 Expert interview method

Interviews are conducted with experts in areas such as topic argumentation, content rationality, and indicator design. These interviews primarily involve: (1) discussions with experts in early childhood physical education, sports education and training, school sports, sports statistics, and measurement concerning topic selection, research content, methodologies, and the choice of indicators. (2) Conversations with kindergarten principals and other administrative staff, which concentrate on the current state of kindergarten physical activities, the practicality of conducting evaluations, and specific challenges related to these assessments. (3) Consultations with front-line kindergarten teachers about the specifics of kindergarten activities, the progress of children's motor development, and the varying levels of difficulty associated with the indicators. These discussions provide a foundation for the development of coordination ability indicators for young children.

### 2.2 Delphi method

To enhance the initially developed indicator system for assessing coordination ability in young children, the Delphi technique was utilized to select the indicators. The expert panel consisted of 16 professionals, each with the rank of associate professor or higher in the fields of early childhood physical education, school sports, and sports pedagogy. Following two rounds of questionnaire completion, the experts feedback was collated, organized, and meticulously analyzed. This procedure resulted in the creation of a refined evaluation framework for the coordination ability of young children.

### 2.3 Testing method

The main data collection of this study was conducted from September to November 2018, covering sampled kindergartens in 16 prefecture-level cities in Shandong Province. A stratified random cluster sampling approach was adopted across 16 prefecture-level cities in Shandong Province. Based on geographical divisions, nine cities were selected from the eastern (Rizhao, Linyi), central (Zibo, Jining, Tai'an, Jinan, and Weifang), and western (Heze, Liaocheng) regions. In each city, two kindergartens were chosen. From each kindergarten, 15 children were randomly selected from both the middle and top classes using a simple random sampling method: children were first numbered by gender within their classes, and then participants were drawn by lot according to gender, ensuring balanced representation (children with known health or developmental conditions that could affect motor or cognitive function were excluded from the study).

A total of 1,080 children were initially tested, including 540 middle-class children (4.0–4.9 years old, 270 boys and 270 girls) and 540 senior-class children (5.0–5.9 years old, 270 boys and 270 girls). After excluding 12 incomplete records, valid data were obtained from 1,068 children, comprising 530 middle-class (264 boys, 266 girls) and 538 top-class children (268 boys, 270 girls).

#### 2.3.1 Pre-test preparations

First, systematic training was provided to the test administrators. The testing team consisted of 12 postgraduate students majoring in Physical Education from three universities, divided into six groups with two members per group specifically responsible for the work of one test station. Prior to data collection, the researchers and test administrators thoroughly studied the assessment protocols, reviewed instructional videos detailing the measurement methods, and standardized the testing procedures and operational details. Additionally, an 8-h intensive training was conducted focusing on the operational standards, scoring criteria, and error avoidance for the 20 indicators, including the study of standardized operational videos, simulated test drills, and on-site Q&A sessions. Second, all assessment forms were prepared with the young children's basic information pre-filled. Meanwhile, the testing equipment and venues were fully inspected to ensure readiness for the formal test.

#### 2.3.2 Participants

The study participants consisted of children aged 4–5.9 years who completed all test items. The assessment of coordination ability in young children is predicated on their physiological maturity. Since 3-year-olds are in the initial stages of fundamental motor skill development and have just entered kindergarten in September, they often experience difficulty adapting to the new environment. Therefore, participant age was calculated using the formula: Age = (Test Date – Birth Date)/12 months. Accordingly, the final sample included children aged 4.0–5.9 years, corresponding to those in the middle and senior kindergarten classes.

#### 2.3.3 Testing procedure

The testing was conducted in two phases: a pilot test and the formal test.

(1) Pilot test

Following the determination of evaluation indicators and testing methods, a pilot test was administered in August 2018 at a kindergarten (Little Dove Kindergarten) to assess the feasibility of the methods, familiarize the team with the testing protocols and equipment, and prepare for the formal data collection. One week after the initial pilot test, the same battery of tests was re-administered to the same group of children to evaluate temporal measurement error and assess the stability of the assessments. Test-retest reliability was determined using Pearson product-moment correlation.

The test-retest method involves administering the same assessment to the same participants after a specified time interval. The reliability of the results is evaluated by calculating the correlation coefficient between the two measurement occasions. The magnitude of the correlation coefficient reflects the degree of measurement consistency. The calculated correlation coefficients (*r*) for all indicators exceeded 0.80, indicating good reliability across all measured dimensions and supporting the use of these indicators for subsequent research (see Appendix Table 1).

Inter-rater reliability was assessed, with evaluators independently scoring/recording test data. Multiple raters were randomly assigned to different children while evaluating the same indicators, using the ICC (2,1) model.

Consistency in scoring was ensured through the following measures:

Pre-test calibration: Prior to formal testing, six groups of evaluators conducted a pre-test on the same five children. Their scoring results were compared, and discrepancies were uniformly calibrated by the principal investigator.

On-site quality control: During the testing process, the principal investigator randomly reviewed 10% of the test data from each site to resolve scoring discrepancies in real time.

Post-hoc verification: Cross-validation was performed on 10% of the sample data to ensure the absence of systematic scoring bias.

The results showed that the ICC (2,1) values of all individual indicators and the composite dimension ranged from 0.864 to 0.945, with no 95% confidence interval including 0, indicating excellent inter-rater consistency. This result verifies the objectivity and stability of the coordination ability assessment tool, providing reliable methodological support for subsequent group comparisons (See Appendix 4).

(2) Formal testing

Kindergartens were selected using a combination of cluster sampling and random sampling methods. Throughout the testing process, the personnel assigned to each specific test item remained consistent, with individuals demonstrating strong sense of responsibility selected to minimize error. To further reduce potential discrepancies arising from variations in evaluators' scoring criteria, the following measures were implemented prior to formal data collection: after all preliminary preparations were completed, two children were invited to participate in a trial run at each testing station following the official protocol. This exercise allowed the testers to gain further familiarity with the procedures, standardize operational details, and identify potential issues. The principal researcher provided on-site supervision and guidance throughout the entire testing process.

### 2.4 Mathematical statistics

The data were collected, organized, and analyzed using descriptive statistics with software such as SPSS 26.0 and AMOS 26.0. The specific procedures are as follows: (1) the structural model of the evaluation indicators for coordination ability in young children was validated using AMOS 26.0 statistical software to assess the fit of the hypothesized model to the measured data. (2) The principal component analysis (PCA) method was used to calculate the weight coefficients for the measurement indicators. (3) The percentile method was utilized to classify evaluation levels for different age groups and to establish the corresponding evaluation criteria. (4) Independent samples *t*-tests were conducted to compare the mean values of coordination ability among different age groups of children in order to determine any significant differences (*P*-values were calculated). An analysis of the development levels of coordination ability in young children was also performed.

## 3 Results

### 3.1 Conceptual definitions

Piaget and Cook (2) argued that coordination originates from the interaction between assimilation and accommodation, as well as the equilibration process, which ultimately leads to schema coordination and a balanced state between distinct schemas. In sports contexts, coordination refers to the body's harmonious organization to resolve movement contradictions, align organs and systems, and accomplish designated motor tasks. Synthesizing physiological, psychological, and sports science perspectives, this study defines coordination ability as a multidimensional motor capability: it relies on the nervous system to integrate motor organs, sensory organs, and internal-external environmental conditions, enabling spatiotemporally precise, adaptive, and efficient movement. Its core dimensions include balance ability, rhythm ability, spatial orientation ability, perceptual judgment ability, limb coordination ability, and limb movement range–all of which collectively support smooth motor execution and energy optimization (see Appendix 3 for details).

The coordination ability of young children (aged 4.0–5.9 years) is a comprehensive skill manifested during fundamental motor skill acquisition (e.g., walking, jumping). It involves the spatiotemporal synchronization of multiple organ systems, encompasses both locomotor and manipulative coordination, and is shaped by physical fitness maturity, prior movement experience, genetics, and family/social environments.

### 3.2 Evaluation of the structural model construction

#### 3.2.1 Theoretical screening of the evaluation elements

Previous research on coordination and related concepts such as coordinative ability and coordination ability has concluded that coordination ability is a multifaceted performance capability. It represents the synergistic functioning of various components and possesses a multidimensional structure. In order to provide a clearer and more precise definition, interviews were conducted with experts in early childhood physical education and kindergarten teachers. These interviews provided valuable insights and suggestions. Furthermore, existing theories regarding the structure and concepts of coordination ability were reviewed, and the characteristics and content of early childhood activities were considered. This informed the preliminary construction of an index system to assess coordination ability in young children. The selection of indicators primarily considered the following aspects: first, the selection process drew on measurement indicators and methods established in previous studies by conducting a preliminary literature review. Second, feasible measurement indicators were identified through expert questionnaires conducted by the research team. Third, pretesting was conducted to ascertain the actual motor skill levels of young children. Based on the results, some indicators with lower feasibility were revised or eliminated.

#### 3.2.2 Expert survey results and analysis

The enthusiasm coefficient of experts, which reflects their attention to and understanding of the research, is generally calculated based on the questionnaire return rate. The authority of experts directly affects the reliability of the questionnaire results and can be quantified by the authority coefficient (*C*_*r*_). The degree of coordination among expert opinions reflects the variation in the evaluation of indicators by the selected experts, and this can be measured by calculating the coefficient of variation (*CV*) and the coordination coefficient (*W*).

Firstly, the return rates of the two rounds of expert questionnaires in this study were above 89%, indicating a high level of attention from the selected expert panel toward this research. Secondly, the statistical results for the authority coefficients show that each evaluation index has an authority coefficient above 0.80, which demonstrates a high degree of expertise within the group of experts. Thirdly, the coefficient of variation (*CV*) for both primary and secondary indicators is less than 0.25, suggesting that after two rounds of screening, the opinions of the experts have converged, and the indicators have been consistently recognized, reflecting a high degree of consensus. Finally, following two rounds of questionnaire surveys, the chi-square values corresponding to the primary and secondary indicators resulted in *P* values less than 0.05, reaching a statistically significant level. This indicates that the expert team selected for this study exhibits a high degree of consensus in their opinions on the assessment of young children's coordination ability, lending credibility to the assessment results.

After expert screening, the evaluation structure for young children's coordination ability has been established, encompassing six key elements and 28 test indicators: balance ability, spatial orientation ability, range of limb movement, rhythmic ability, limb coordination ability, and perceptual judgment ability. This model will undergo confirmatory factor analysis to provide a robust foundation for evaluating the coordination ability of young children. These revisions maintain the original meaning and tone while enhancing the grammatical precision and readability of the text.

#### 3.2.3 Validity and reliability test

The measurement indicators selected for this study are primarily derived from existing domestic and international research literature. They have been established by integrating the research background with practical problems, grounded in the underlying concept. Furthermore, the questionnaire design has incorporated expert insights, with 75% of the experts rating the importance of these indicators as important or higher, indicating a strong content validity.

The structural validity was primarily assessed using exploratory factor analysis (EFA). The EFA results indicated that eight indicators, including perception of rhythm speed, perception of rhythm intensity, catching a ball, directional throwing, side roll, forward bend from a standing position, single-leg hopping, and two-leg hopping, exhibited factor loadings significantly below 0.5. This suggests a deficiency in convergent validity, meaning these indicators did not measure the intended construct as expected. Additionally, experts observed in the questionnaire that the indicators of rhythm intensity and speed under the rhythm reproduction dimension primarily reflect the child's level of attention and memory, while the indicators of spatial movement accuracy are closely related to the child's regular ball sports training. Furthermore, side roll is particularly challenging for younger children. Following a thorough analysis, the decision was made to exclude these indicators from the assessment. Additionally, the indicator high knee lifts in place had high loadings on two factors, suggesting a cross-factor phenomenon. Due to its complexity and the challenges it presented during testing, this movement was also excluded from the analysis. Following a subsequent factor analysis, the revised indicators all demonstrated factor loadings above 0.5, satisfying the fundamental criteria for reliable analysis.

The reliability of this study was assessed using Cronbach's alpha coefficient. The findings revealed that the reliability for each dimension generally exceeded 0.6, reflecting a good level of reliability. Specifically, the reliability of the directional ability dimension was 0.593, which is nearly at the acceptable threshold of 0.60. Upon eliminating the straight-leg raise test from the limb movement dimension, the reliability of this dimension improved, increasing from 0.578 to 0.734. Consequently, this indicator was removed from the study. McDonald's Omega was also calculated to assess reliability, yielding a coefficient of 0.893, which indicates high internal consistency. The procedure was as follows: first, a factor analysis was run to obtain the factor loadings. The key metrics were then derived, including the sum of the loadings (ΣLoading), the sum of squared loadings (Σ Loading^2^), and the sum of the uniqueness. Finally, these values were substituted into the formula for McDonald's Omega: ω = (Σ Loading)^2^/[(Σ Loading)^2^ +Σ Uniqueness].

#### 3.2.4 Validation of coordination evaluation model

**Figure 1:**
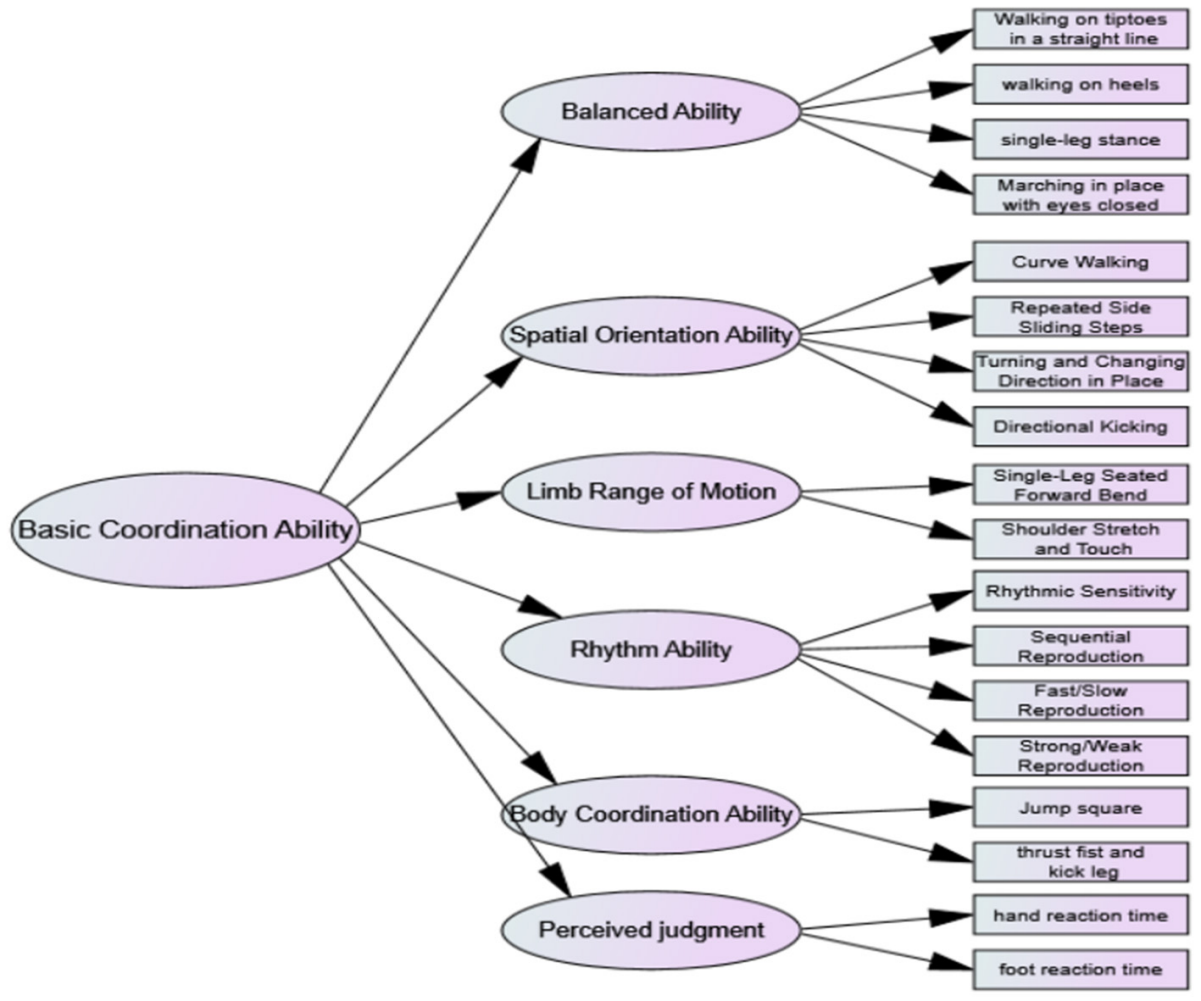
Structural model of coordination ability evaluation for young children.

Confirmatory factor analysis primarily examines the degree of similarity between the hypothesized model and the measurement indicators. The revised model demonstrates ideal fit indices: χ^2^/df = 1.564, GFI, CFI, TLI > 0.90, and RMSEA = 0.046. These results confirm a good fit between the theoretical model and the measurement data, supporting the acceptance of the hypothesized model (Table 1). Through confirmatory factor analysis, we obtained the evaluation structure model for the coordination ability in young children (Figure 1). The model consists of structural elements such as the balance ability, the spatial orientation ability, the rhythmic ability, the perceptual judgment ability, the limb coordination ability, and the range of limb movement. Each of these elements is composed of 2–4 test indicators, which also serve as the main factors for evaluating the level of coordination ability in young children.

**Table 1:** First-order confirmatory factor analysis fitting test results (*n* = 268).

**Fit indices**	**χ^2^**	**DF**	**χ^2^/df**	**RMSEA**	**GFI**	**AGFI**	**CFI**	**TLI**
Before the correction	232.621	122	1.907	0.058	0.916	0.882	0.906	0.920
After the correction	187.649	120	1.564	0.046	0.932	0.903	0.961	0.951

### 3.3 Weight determination

The test sample in this study comprises children from the middle and senior classes of kindergartens. A multifactorial analysis of variance reveals that gender exerts a negligible influence on the selected test indicators, whereas age significantly affects them. Consequently, the sample has been divided into two age groups according to age: the 4-year-old group (aged 4.0–4.9 years) and the 5-year-old group (aged 5.0–5.9 years).

The data were analyzed using multifactorial analysis of variance (ANOVA) in SPSS software. Levene's test for homogeneity of variance yielded significance levels (*P*) greater than 0.05 for all items, indicating equal variances across groups and satisfying the assumptions for ANOVA. Tests of main effects showed that gender had no significant effect on the indicators (*P* > 0.05), suggesting no statistically significant difference in the measured indicators based on the children's gender. In contrast, the main effect of age was significant for all indicators except seated single-leg forward bend, eyes-closed marching in place, and single-leg stance, as determined by class-based age grouping (*P* < 0.05). The detailed results of the age effect tests are provided in Appendix Table 2.

Therefore, for the evaluation of coordination ability in young children, participants were divided into age groups based on class: a 4-year-old group (middle class, 4.0–4.9 years) and a 5-year-old group (upper class, 5.0–5.9 years). Children aged 6 years or above were not included. This grouping approach was adopted for two main reasons: first, it reflects age-related differences in the development of coordination ability by focusing on distinct developmental stages; second, it ensures practical feasibility during testing. Based on the analysis of all indicators of coordination ability, the study concludes that children should be grouped into the 4-year-old group (middle class, 4.0–4.9 years) and the 5-year-old group (upper class, 5.0–5.9 years). This grouping is also consistent with the age categories recommended in the “3–6 Year Old Children Learning and Development Guide.”

Given the need to incorporate expert judgment in determining the relative importance of each coordination ability indicator, the Analytic Hierarchy Process (AHP) was employed to construct pairwise comparison matrices, transforming qualitative expert opinions into quantitative weights. This approach compensates for the limitations of purely data-driven methods, which often overlook practical experience.

To address the correlations among test indicators, Principal Component Analysis (PCA) was applied to reduce dimensionality and extract principal components. The variance contribution rate of each component served as the basis for weight allocation, thereby minimizing the influence of indicator redundancy on the weighting results and reflecting the inherent structural characteristics of the data. To account for the dispersion of each indicator (i.e., an indicator's ability to differentiate among children's skill levels), the coefficient of variation (CV) method was used, calculated as the standard deviation divided by the mean. Indicators with higher CV values were assigned greater weights, ensuring that the weighting scheme considered the objective discriminative power of the data. These three methods complement each other across three dimensions: “expert judgment,” “data structure,” and “discriminative ability.” Together, they enhance the comprehensiveness and rationality of the weighting results and mitigate the limitations of relying on any single method.

#### 3.3.1 Weights of primary indicators

##### 3.3.1.1 Construction of the judgment matrix for indicators

The primary comparison involves six factors (*C*_1_, *C*_2_, *C*_3_, *C*_4_, *C*_5_, *C*_6_) in relation to their influence on the higher-level element *O* (coordination ability in young children), which has led to the creation of the judgment matrix *C*:


$$C=|\begin{array}{cccccc} 1 & 0.33 & 0.33 & 1/3 & 0.14 & 1\\ 3 & 1 & 0.33 & 1 & 0.14 & 3\\ 3 & 3 & 1 & 1 & 0.33 & 3\\ 3 & 1 & 1 & 1 & 0.20 & 3\\ 7 & 7 & 3 & 5 & 1 & 7\\ 1 & 0.33 & 0.33 & 0.33 & 0.14 & 1 \end{array}|$$

##### 3.3.1.2 Calculation of the eigenvector and indicator weights

The sum method is employed to calculate the eigenvalues of the comparison matrix, with the following steps:

(1) Summation of matrix columns
$$C_{1}=|\begin{array}{cccccc} 18 & 12.66 & 5.99 & 8.66 & 1.95 & 18 \end{array}|$$
(2) Normalization for each column:
  Its formula is: $D_{ij}=\frac{C_{ij}}{\sum C_{ij}}$
  The matrix C is normalized according to the formula to obtain matrix D:
$$\text{D}=|\begin{array}{cccccc} 0.06 & 0.03 & 0.06 & 0.04 & 0.07 & 0.06\\ 0.17 & 0.08 & 0.06 & 0.12 & 0.07 & 0.17\\ 0.17 & 0.24 & 0.17 & 0.12 & 0.17 & 0.17\\ 0.17 & 0.08 & 0.17 & 0.12 & 0.10 & 0.17\\ 0.39 & 0.55 & 0.50 & 0.58 & 0.51 & 0.39\\ 0.06 & 0.03 & 0.06 & 0.04 & 0.07 & 0.06 \end{array}|$$
(3) Sum for each row to derive the feature vector
$${\text{D}}_{1}=|\begin{array}{cccccc} 0.30 & 0.65 & 1.02 & 0.80 & 2.92 & 0.30 \end{array}{|}^{T}$$
(4) The indicator weights are calculated by normalizing the eigenvector D_1_, using the following formula:
  公式为: $W_{i}=\frac{D_{j}}{\sum D_{j}}$
$$W=|\begin{array}{cccccc} 0.05 & 0.11 & 0.17 & 0.13 & 0.49 & 0.05 \end{array}{|}^{T}$$

##### 3.3.1.3 Matrix consistency test

(1) Calculate the maximum feature root
$$\lambda_{\max}=\frac{\sum {\left(CW\right)}_{i}}{nW_{i}}$$
  In the formula, *CW*represents the product of matrix *C* and vector *W*. The product can be computed using the matrix multiplication function mmult() in Excel. $CW=|\begin{array}{cccccc} 0.30 & 0.67 & 1.09 & 0.81 & 3.13 & 0.30 \end{array}{|}^{T}$,λ_max_ = 6.20°
(2) Calculate the consistency index.
$$\text{formula}:C.I.=\frac{\lambda_{max}-n}{n-1},\text{得}:C.I.=\frac{6.20-6}{5}=0.04$$
(3) Calculate the random consistency ratio
$$\begin{array}{l} C.R.=\frac{C.I.}{R.I.}\left(R.I.is\;the\;constant\right),\\ \text{get}:C.R.=0.04/1.24=0.03 \end{array}$$

*C*.*R*. < 0.1 indicates that the matrix has consistency, and the obtained weights are valid. Therefore, the weights for the primary indicators of coordination ability in young children are determined (Table 2).

**Table 2:** Weights of the primary indicators for children's coordination ability.

**Level 1 indicators**	**Balanced ability**	**Spatial orientation ability**	**Rhythm ability**	**Perceived judgment**	**Body coordination ability**	**Limb range of motion**
Weight coefficient	0.05	0.11	0.17	0.13	0.49	0.05

#### 3.3.2 Secondary index weight

##### 3.3.2.1 Determination of the balance ability index weight

The principal component analysis (PCA) method was utilized to calculate the weights. For instance, when examining the balance ability indicators of the 4-year-old group, the Kaiser–Meyer–Olkin (KMO) statistic was initially assessed at 0.721, suggesting that PCA is appropriate for determining the weights. As depicted in Table 3, the eigenvalues for the first two principal components exceed 1. Moreover, the cumulative variance contribution rate of these two components amounts to 82.963%, which surpasses the 80% threshold. Consequently, these two principal components can effectively substitute the original set of four indicators to reflect the balance ability information.

**Table 3:** Explained total variance.

**Component**	**Initial eigenvalue**		
	**Characteristic root**	**Variance percentage**	**Accumulate %**
1	1.887	47.185	47.185
2	1.231	35.778	82.963
3	0.897	12.414	95.377
4	0.185	4.623	100.000

Table 4 presents the component matrix, displaying the loading values of the original indicators on the first and second principal components. For instance, the first principal component has a loading value of 0.936 for Walking on tiptoes in a straight line and 0.932 for the heel goes straight.

**Table 4:** Component matrix table.

**Metric**	**Ingredient**	
	**1**	**2**
x_1_	0.936	−0.028
x_2_	0.932	−0.094
x_3_	−0.080	0.880
x_4_	0.370	0.497

X_1_, walking on tiptoes in a straight line; X_2_, heel goes straight; X_3_, stand on one leg; X_4_, marching in place with eyes closed.

Firstly, the coefficients of each indicator in the linear combination of the two principal components are calculated (Table 5). These coefficients are obtained by dividing the loading values in Table 4 by the square root of the corresponding eigenvalues in the first column of Table 3. For example, the coefficient of X1 under the first principal component is calculated as follows: $0.936/\sqrt{1.887}\approx$ 0.681, therefore, the coefficients of each indicator in the linear combination of the two principal components are obtained (Table 5), and the linear combinations of the two principal components are as follows:


$$\begin{array}{l} F_{1}={0.681\text{x}}_{1}+0.678{\text{x}}_{2}-0.058{\text{x}}_{3}+0.269{\text{x}}_{4}\\ F_{2}=-0.025{\text{x}}_{1}-0.084{\text{x}}_{2}+0.793{\text{x}}_{3}+0.447{\text{x}}_{4} \end{array}$$

**Table 5:** Table of coefficients for indicators in the linear combination of main components.

**Metric**	**The 1st principal component coefficient**	**Second principal component coefficient**
x_1_	0.681	−0.025
x_2_	0.678	−0.084
x_3_	−0.058	0.793
x_4_	0.269	0.447

X_1_, walking on tiptoes in a straight line, X_2_, walking on heels, X_3_, single-leg stance, X_4_, marching in place with eyes closed.

Secondly, the variance contribution rates of the principal components are calculated. The indicator coefficients are weighted by the variance contribution rates of the two principal components and then averaged. For example, the coefficient of X1 is calculated as follows: $\frac{0.681\times 47.185-0.025\times 35.778}{47.185+35.778}=$ 0.377. The coefficients in the comprehensive scoring model are obtained (x_1_ = 0.377, x_2_ = 0.349, x_3_ = 0.309, x_4_ = 0.346), and the comprehensive scoring model is as follows:


$$Y={0.377\text{x}}_{1}+0.349{\text{x}}_{2}+0.309{\text{x}}_{3}+0.346{\text{x}}_{4}$$

Finally, the indicators are normalized to obtain the indicator weights. The same method is used to calculate the weights of the balance ability indicators for the 5-year-old group (Table 6).

**Table 6:** Index weights of children's balance ability indicators.

**Group**	**Metric**	**Coefficients**	**Weight**
4-yearyear-old group	x_1_	0.377	0.273
	x_2_	0.349	0.253
	x_3_	0.309	0.224
	x_4_	0.346	0.250
5-yearyear-old group	x_1_	0.419	0.287
	x_2_	0.403	0.276
	x_3_	0.271	0.186
	x_4_	0.368	0.251

X_1_, walking on tiptoes in a straight line; X_2_, walking on heels; X_3_, single-leg stance; X_4_, marching in place with eyes closed.

##### 3.3.2.2 Index weight of rhythmic ability, spatial orientation ability, and limb range of motion

According to the principal component analysis, the weights of rhythm ability, spatial orientation ability and limb range of motion in the 4-year-old group and 5-year-old group were also obtained (Tables 7, 8).

**Table 7:** Index weights of children's indicators in the 4-year-old group.

**Dimension**	**Metric**	**Coefficients**		**Coefficients in the composite score model**	**Indicator weight**
		**Principal Components 1** *F*_1_	**Principal Components 2** *F*_2_		
Rhythm ability	x_5_	0.347	0.788	0.473	0.317
	x_6_	0.568	−0.311	0.317	0.213
	x_7_	0.529	−0.455	0.248	0.166
	x_8_	0.526	0.275	0.454	0.304
Spatial orientation ability	x_9_	0.624	0.156	0.418	0.332
	x_9_	0.599	0.047	0.356	0.284
	x_10_	0.020	0.702	0.320	0.255
	x_11_	0.442	−0.194	0.163	0.129
Limb range of motion	x_3_	0.652	−0.085	0.353	0.277
	x_14_	0.654	−0.064	0.362	0.285
	x_15_	−0.039	0.776	0.292	0.230
	x_16_	−0.082	0.769	0.264	0.208

X_5_, rhythmic sensitivity; X_6_, sequential reproduction; X_7_, fast/slow reproduction; X_8_, strong/weak reproduction; X_9_, curve walking; X_10_, repeated side sliding steps; X_11_, Turning and changing direction in place; X_12_, directional kicking; X_13_, single-leg seated forward bend (left); X_14_, single-leg seated forward bend (Right); X_15_, shoulder stretch and touch (left); X_16_, Shoulder stretch and touch (right).

**Table 8:** Index weights of children's indicators in the 5-year-old group.

**Dimension**	**Metric**	**Coefficients**		**Coefficients in the composite score model**	**Indicator weight**
		**Principal Components 1** *F*_1_	**Principal Components 2** *F*_2_		
Rhythm ability	x_5_	0.383	0.876	0.494	0.310
	x_6_	0.550	−0.304	0.358	0.224
	x_7_	0.527	−0.371	0.325	0.204
	x_8_	0.523	0.05180518	0.417	0.262
Perceived judgment	x_9_	0.536	0.723	0.615	0.297
	x_10_	0.615	0.521	0.575	0.278
	x_11_	0.535	0.590	0.558	0.269
	x_12_	0.298	0.358	0.323	0.156
Limb range of motion	x_13_	0.632	−0.121	0.364	0.307
	x_14_	0.631	−0.123	0.363	0.306
	x_15_	−0.041	0.820	0.265	0.224
	x_16_	−0.137	0.792	0.193	0.163

X_5_, rhythmic sensitivity; X_6_, sequential reproduction; X_7_, fast/slow reproduction; X_8_, strong/weak reproduction; X_9_, curve walking; X_10_, repeated side sliding steps; X_11_, Turning and changing direction in place; X_12_, directional kicking; X_13_, single-leg seated forward bend (left); X_14_, single-leg seated forward bend (right); X_15_, shoulder stretch and touch (left); X_16_, shoulder stretch and touch (right).

##### 3.3.2.3 The index weight of limb coordination ability and perceptual judgment ability

The coefficient of variation method is used to calculate the index weight of limb coordination ability and perceptual judgment ability. The calculation formula is:


$$W_{i}=\frac{U_{i}}{\overset{n}{\underset{i=1}{\sum }}U_{i}}$$

The coefficient of variation and the corresponding weights of each index are shown in Table 9.

**Table 9:** Index weights of children's limb coordination, perceptual judgment ability.

**Dimension**	**Class**	**Metric**	**Mean**	**Standard deviation**	**Coefficient of variation**	**Weight**
Body coordination ability	4 yearYears old group	x_17_	11.726	3.965	0.338	0.358
		x_18_	20.87	12.665	0.607	0.642
	5 yearYears old group	x_17_	8.840	2.366	0.268	0.345
		x_18_	28.56	14.499	0.508	0.655
Perceived judgment	4 yearYears old group	x_19_	35.400	5.481	0.155	0.614
		x_20_	45.454	4.423	0.097	0.386
	5 YearYears old group	x_19_	33.921	6.308	0.186	0.598
		x_20_	37.592	4.702	0.125	0.402

X_17_, jump square; X_18_, thrust fist and kick leg; X_19_, hand reaction time; X_20_, foot reaction time.

#### 3.3.3 Evaluation index system is established

Based on the indicator weights, the test indicators under the primary indicators were normalized, and the final weights of the indicators were calculated. The evaluation index system for coordination ability in young children was ultimately determined, with the results shown in Table 10.

**Table 10:** Evaluation index system for the coordination ability.

**Level 1 indicators**	**Weight**	**Evaluating indicator**	**4 years old group**		**5 years old group**	
			**Indicator weight**	**The final weight**	**Indicator weight**	**The final weight**
Balanced ability	0.05	x_1_	0.273	0.014	0.287	0.014
		x_2_	0.253	0.013	0.276	0.014
		x_3_	0.224	0.011	0.186	0.009
		x_4_	0.250	0.013	0.251	0.013
Rhythm ability	0.17	x_5_	0.317	0.05	0.310	0.053
		x_6_	0.213	0.04	0.224	0.038
		x_7_	0.166	0.03	0.204	0.035
		x_8_	0.304	0.05	0.262	0.045
Spatial orientation ability	0.11	x_9_	0.332	0.04	0.297	0.03
		x_10_	0.284	0.03	0.278	0.03
		x_11_	0.255	0.03	0.269	0.03
		x_12_	0.129	0.01	0.156	0.02
Limb range of motion	0.05	x_13_	0.277	0.014	0.307	0.015
		x_14_	0.285	0.014	0.306	0.015
		x_15_	0.230	0.012	0.224	0.011
		x_16_	0.208	0.010	0.163	0.008
Body coordination ability	0.49	x_17_	0.358	0.18	0.345	0.17
		x_18_	0.642	0.31	0.655	0.32
Perceived judgment	0.13	x_19_	0.614	0.08	0.598	0.08
		x_20_	0.386	0.05	0.402	0.05

X_1_, walking on tiptoes in a straight line; X_2_, walking on heels; X_3_, single-leg stance; X_4_, marching in place with eyes closed; X_5_, rhythmic sensitivity; X6, sequential reproduction; X_7_, fast/slow reproduction; X_8_, strong/weak reproduction; X_9_, curve walking; X_10_, repeated side sliding steps; X_11_, turning and changing direction in place; X_12_, directional kicking; X_13_, single-leg seated forward bend (left); X_14_, single-leg seated forward bend (right); X_15_, shoulder stretch and touch (left); X_16_, shoulder stretch and touch (right); X_17_, jump square; X_18_, thrust fist and kick leg; X_19_, hand reaction time; X_20_, foot reaction time.

### 3.4 Establishment of the evaluation criteria for coordination ability in young children

#### 3.4.1 Development of the evaluation criteria for individual indicators

The evaluation criteria for individual indicators of coordination ability in young children were developed based on the percentile method. We adopted a five-level scoring system. In alignment with the approach used in the National Physical Fitness Testing Standard, scores were not assigned to values below the P3 percentile to prompt teachers and parents to focus more on enhancing the coordination ability of young children (16).

Firstly, the percentile scores for each indicator of coordination ability in young children were calculated. Secondly, using the percentile score table, the indicators were categorized into five distinct levels. The corresponding scores for each level were then determined as a percentage (with levels corresponding to scores of 20, 40, 60, 80, and 100). Each indicators weighted score was derived by multiplying its weight by the corresponding level score. Finally, after compiling the data, five-level scoring tables for each indicator at various age stages were created (refer to Tables 11, 12). These tables detail the evaluation indicators, the five levels, and the associated weighted scores.

**Table 11:** Scores of children's coordination ability in the 4-year-old group.

**Index x**	**The fifth level 20 points**	**The fourth level 40 points**	**The third level 60 points**	**The second level 80 points**	**The first level 100 points**
x_1_/s	8.67–11.24	6.36–8.66	4.42–6.35	3.16–4.41	<3.16
Score	0.28	0.56	0.84	1.12	1.4
x_2_/s	8.98–12.50	6.85–8.97	5.36–6.84	3.50–5.35	<3.50
Score	0.26	0.52	0.78	1.04	1.3
x_3_/s	2.22–3.75	3.76–7.94	7.95–13.48	13.49–28.65	>28.65
Score	0.22	0.44	0.66	0.88	1.1
x_4_/s	3.40–6.83	6.84–11.25	11.26–19.49	19.50–36.04	>36.04
Score	0.26	0.52	0.78	1.04	1.3
x_5_	1.7–2.6	2.7–3.6	3.7–4.2	4.3–4.9	5
Score	1	2	3	4	5
x_6_	1	2	3	4	5
score	0.8	1.6	2.4	3.2	4
x_7_	1	2	3	4	5
Score	0.6	1.2	1.8	2.4	3
x_8_	1	2	3	4	5
Score	1	2	3	4	5
x_9_/s	8.72–9.81	7.18–8.71	6.35–7.17	5.59–6.34	<5.59
Score	0.8	1.6	2.4	3.2	4
x_10_	11–13	14–18	19–21	22–24	>24
score	0.6	1.2	1.8	2.4	3
x_11_/s	22.43–24.29	18.51–22.42	15.86–18.50	13.28–15.85	<13.28
Score	0.6	1.2	1.8	2.4	3
x_12_	1–2	3–4	5–6	7	>7
score	0.2	0.4	0.6	0.8	1
x_13_/cm	−2.79 to 1.44	1.45–3.99	4–7.99	8–11.99	>11.99
Score	0.28	0.56	0.84	1.12	1.4
x_14_/cm	−2.30 to 1.40	1.40–3.99	4–7.99	8–12.99	>12.99
Score	0.28	0.56	0.84	1.12	1.4
x_15_/cm	25.1–27	20.1–25	16.1–20	11.1–16	<11.1
score	0.24	0.48	0.72	0.96	1.2
x_16_/cm	24.1–26.8	19.1–24	15.1–19	10.1–15	<10.1
Score	0.2	0.4	0.6	0.8	1
x_17_/s	17.48–21.84	12.62–17.47	9.81–12.61	7.48–9.80	<7.48
Score	3.6	7.2	10.8	14.4	18
x_18_	2–4	5–13	14–25	26–38	>38
score	6.2	12.4	18.6	24.8	31
x_19_/cm/s	44.20–49.37 0.32	37.01–44.19 0.30–0.31	33.31–37 0.28–0.29	29.14–33.30 0.26–0.27	<29.13 <0.25
Score	1.6	3.2	4.8	6.4	8
x_20_/cm/s	50.01–54	47.61–50	42.54–47.60	39.21–42.53	<39.21
	0.33	0.32	0.31	0.29–0.30	<0.28
Score	1	2	3	4	5

X_1_, walking on tiptoes in a straight line; X_2_, walking on heels; X_3_, single-leg stance; X_4_, marching in place with eyes closed; X_5_, rhythmic sensitivity; X_6_, sequential reproduction; X_7_, fast/slow reproduction; X_8_, strong/weak reproduction; X_9_, curve walking; X_10_, repeated side sliding steps; X_11_, turning and changing direction in place; X_12_, directional kicking; X_13_, Single-leg seated forward bend (left); X_14_, single-leg seated forward bend (right); X_15_, shoulder stretch and touch (left); X_16_, shoulder stretch and touch (right); X_17_, jump square; X_18_, thrust fist and kick leg; X_19_, hand reaction time; X_20_, foot reaction time.

**Table 12:** Scores of children's coordination ability in the 5-year-old group.

**Index X**	**The fifth level 20分**	**The fourth level 40分**	**The third level 60分**	**The second level 80分**	**The first level 100分**
x_1_/s	8.49–10.32	6.00–8.48	4.37–5.99	2.88–4.36	<2.88
Score	0.28	0.56	0.84	1.12	1.4
x_2_/s	8.53–11.70	6.21–8.52	5.11–6.20	3.48–5.10	<3.48
Score	0.28	0.56	0.84	1.12	1.4
x_3_/s	2.70–4.64	4.65–12.04	12.05–22.96	22.97–47.99	>47.99
Score	0.18	0.36	0.54	0.72	0.9
x_4_/s	5.31–7.29	7.30–11.55	11.56–20.59	20.60–39.47	>39.47
Score	0.26	0.52	0.78	1.04	1.3
x_5_	2.7–2.9	3.0–3.9	4.0–4.6	4.7–4.9	5
Score	1.06	2.12	3.18	4.24	5.3
x_6_	1	2	3	4	5
Score	0.76	1.52	2.28	3.04	3.8
x_7_	1	2	3	4	5
Score	0.7	1.4	2.1	2.8	3.5
x_8_	1	2	3	4	5
Score	0.9	1.8	2.7	3.6	4.5
x_9_/s	7.92–8.66	6.67–7.91	6.01–6.66	5.29–6.00	<5.29
Score	0.6	1.2	1.8	2.4	3
x_10_	13–17	18–20	21–23	24–26	>26
Score	0.6	1.2	1.8	2.4	3
x_11_/s	18.33–21.17	15.31–18.32	13.11–15.30	10.59–13.10	<10.59
Score	0.6	1.2	1.8	2.4	3
x_12_	2–3	4–5	6–7	8	>8
Score	0.4	0.8	1.2	1.6	2
x_13_/cm	−9.5 to 1.49	−1.50 to 2.99	3–7.19	7.2–14.1	>14.1
Score	0.3	0.6	0.9	1.2	1.5
x_14_/cm	−7.6 to 1.50	−1.50 to 2.99	3–7.29	7.3–13.99	>13.99
Score	0.3	0.6	0.9	1.2	1.5
x_15_/cm	23.1–29	18.1–23	13.9–18	8.7–13.8	<8.7
Score	0.22	0.44	0.66	0.88	1.1
x_16_/cm	22.1–27	17.1–22	13.1–17	8.1–13	<8.1
Score	0.16	0.32	0.48	0.64	0.8
x_17_/s	11.97–14.20	9.48–11.96	7.61–9.47	6.04–7.60	<6.04
Score	3.4	6.8	10.2	13.6	17
x_18_	4–9	10–20	21–34	35–47	>47
Score	6.4	12.8	19.2	25.6	32
x_19_/cm/s	41.45–47.20	35.96–41.44	31.51–35.95	26.51–31.50	<26.51
	0.30–0.31	0.28–0.29	0.26–0.27	0.24–0.25	<0.23
Score	1.6	3.2	4.8	6.4	8
x_20_/cm/s	44.26–50	39.01–44.25	35.01–39	31.01–35	<30.01
	0.31–0.32	0.30	0.28–0.29	0.26–0.27	<0.25
Score	1	2	3	4	5

X1, walking on tiptoes in a straight line; X2, walking on heels; X3, single-leg stance; X4, marching in place with eyes closed; X5, rhythmic sensitivity; X6, sequential reproduction; X7, fast/slow reproduction; X8, strong/weak reproduction; X9, curve walking; X10, Repeated side sliding steps; X11, turning and changing direction in place; X12, directional kicking; X13, single-leg seated forward bend (left); X14, single-leg seated forward bend (right); X15, Shoulder stretch and touch (left); X16, shoulder stretch and touch (right); X17, jump square; X18, thrust fist and kick leg; X19, hand reaction time; X20, foot reaction time.

#### 3.4.2 Development of the comprehensive evaluation criteria

Based on the individual indicators of coordination ability in young children, the weighted sum method is used for calculation, with the formula being: comprehensive score (total score) = ∑(P_x_×W_x_). Here, P_x_ represents the score of individual indicator x, and W_x_ represents the weight of indicator x. The comprehensive evaluation tables for coordination ability in the 4-year-old group and the 5-year-old group are shown in Tables 13, 14.

**Table 13:** Comprehensive score of children's coordination ability in the 4-year-old group (weighted; *n* = 426).

**Metric**	**Level 1 (Excellent)**	**Level 2 (Good)**	**Level 3 (qualified)**	**Level 4 (unqualified)**
Balanced ability	≥4.07	3.33–4.06	2.30	<2.30
Rhythm ability	≥16	14–15.99	6.40–13.99	<6.40
Spatial orientation ability	≥8.80	7.20–8.19	5.00–7.19	<5.00
Limb range of motion	≥4.20	3.52	2.08	<2.08
Body coordination ability	≥39.98	33–39.97	19.65	<19.65
Perceived judgment	≥11.40	9.80–11.39	6.20	<6.20
Comprehensive score	≥76.05	67.01–76.05	51.40–67.0	<51.40
The theoretical percentage	10	25	50	15
Percentile	P_90_ or more	P_65_-P_90_	P_15_-P_65_	P_15_ below

**Table 14:** Comprehensive score of children's coordination ability in the 5-year-old group (weighted) (*n* = 416).

**Metric**	**Level 1 (Excellent)**	**Level 2 (Good)**	**Level 3 (qualified)**	**Level 4 (unqualified)**
Balanced ability	≥3.81	3.21–3.80	2.20–3.20	<2.20
Rhythm ability	≥16.05	14.58–16.04	8.70–14.57	<8.70
Spatial orientation ability	≥8.20	7.11–8.19	4.80–7.10	<4.80
Limb range of motion	≥4.08	3.31–4.07	2.10–3.30	<2.10
Body coordination ability	≥42.1	32.8–42.0	19.5–32.7	<19.5
Perceived judgment	≥10.5	8.5–10.4	4.0–8.4	<4.0
Comprehensive score	≥75.56	67.95–75.55	50–67.94	<50
The theoretical percentage	10	25	50	15
Percentile	P_90_ or more	P_65_-P_90_	P_15_-P_65_	P_15_ below

The evaluation model formula for coordination ability in young children is:

4 yearYears old group (4.0–4.9) = 0.014x_1_ + 0.013x_2_ + 0.011x_3_ + 0.013x_4_ + 0.05x_5_ + 0.04x_6_ + 0.03x_7_ + 0.05x_8_ + 0.04x_9_ + 0.03 (x_10_ + x_11_) + 0.01x_12_ + 0.014 (x_13_ + x_14_)+ 0.012x_15_ + 0.01x_16_ + 0.18x_17_ + 0.31x_18_ + 0.08x_19_ + 0.05x_20_

5 yearYears old group (5.0–5.9) = 0.014 (x_1_ + x_2_)+ 0.009x_3_ + 0.013x_4_ + 0.053x_5_ + 0.038x_6_ + 0.035x_7_ + 0.045x_8_ + 0.03 (x_9_ + x_10_ + x_11_) + 0.02x_12_ + 0.015 (x_13_ + x_14_)+ 0.011x_15_ + 0.008x_16_ + 0.17x_17_ + 0.32x_18_ + 0.08x_19_ + 0.05x_20_

#### 3.4.3 Reliability validation of the comprehensive evaluation standard

To assess the reliability of the newly established evaluation standard, a validation procedure was conducted by partitioning the sample data into a modeling set (80%) and a testing set (20%). The testing set (*n* = 226) was then subjected to a back-testing procedure to calculate the classification error rate. A lower error rate indicates higher reliability of the scoring standard, as it reflects better agreement between the model's predictions and the theoretical classification percentages.

The back-testing procedure was as follows:

(1) Data preparation: the raw test data from the sampled population were compiled.
(2) Score assignment: for each subject in the testing set, individual test performance metrics were mapped to their corresponding component scores using the established Individual Score Sheet.
(3) Composite score calculation and level classification: the composite score for each subject was computed by summing the weighted scores of all individual metrics, according to the predefined weighting scheme. This final composite score was then classified into the appropriate proficiency level based on the comprehensive scoring table.

The results of this classification for the test set are summarized in Table 15. The analysis revealed that the back-tested classifications for both the 4-year-old and 5-year-old groups aligned well with the theoretical percentages. The overall misclassification rate (error rate) was below 5%, suggesting a good fit.

**Table 15:** List of back-testing for comprehensive evaluation of coordination ability.

**Class**	**Level 1 (Excellent)**	**Level II (Good)**	**Level 3 (qualified)**	**Level 4 (unqualified)**	**Total**
Number/person in the 4-year-old group	11	24	53	16	104
Percentage%	10.37	23.97	50.51	15.15	100
Error rate%	+3.7	−4.1	+1.0	+1.0	
Number/person in the 5-year-old group	12	32	60	18	122
Percentage%	9.8	26	49.2	14.8	100
Error rate%	−2	+4	−1.6	−1.3	

However, it is important to note a key methodological limitation: the reliability assessment was based on a single, static split of the data. To quantitatively express the uncertainty associated with the observed error rate, we calculated its 95% confidence interval (CI) using the Wilson score interval method, which is suitable for binomial proportions. The overall error rate was 2.3% (95% CI: 1.0%−4.1%). While the point estimate of the error rate is below 5%, the width of the confidence interval indicates the precision of this estimate.

Based on the point estimate of the error rate being within an acceptable threshold (<5%), it is concluded that the comprehensive evaluation standard for children's coordination ability demonstrates preliminary reliability. The established standard is therefore considered valid for initial use.

### 3.5 Children's coordination ability development characteristics

The development level of basic motor skills and coordination ability in early childhood significantly impacts children's participation in sports and their lifelong health. Therefore, it is crucial to closely monitor children's participation in activities and accurately assess the development of their coordination ability. Utilizing the measured data, this section employs newly established evaluation criteria for the coordination ability of young children to analyze the patterns associated with age and gender. This analysis is intended to provide a foundation for the subsequent design of the curriculum. The study primarily utilizes independent samples *t*-tests alongside effect size to compare the performance of children across different age groups. This analysis investigates gender differences in specific indicators, structural components, as well as in overall scores. The effect size is employed to quantify the magnitude of the difference between the two samples (17). Cohen's *d* value is used to determine the extent of gender differences, with a *d* value of 0.8 or higher denoting a large effect size, a *d* value of 0.5 indicating a medium effect size, and a *d* value of 0.2 or lower suggesting a small effect size.

#### 3.5.1 The development level of coordination ability in 4-year-old children (middle class)

Tables 16, 17 present the gender differences in the indicators of coordination ability, as well as the 20 individual test indicators, in young children. The comprehensive evaluation results of coordination ability indicate that girls scored higher than boys, with a significant difference in the total score (*P* < 0.01). Among the component element indicators, only perceptual judgment ability was higher in boys than in girls, but this difference was not statistically significant. All other indicators were lower in boys than in girls. Specifically, the range of limb movement and limb coordination ability were higher in girls than in boys, with significant differences (*P* < 0.01). In terms of effect size, the differences in the indicators with significant differences and the comprehensive score were small to medium (*d* = 0.39–0.47).

**Table 16:** Gender differences in the development of children's coordination ability (4-year-old) ($\bar{X}\pm S$).

**Group**	**Female**	**Male**	***P*-value**	***d***
**Single metric score (unweighted)**				
X_1_	61.21 ± 25.84	60 ± 22.49	0.719	0.05
X_2_	61.41 ± 23.26	58.13 ± 23.88	0.319	0.14
X_3_	64.65 ± 26.16	54.58 ± 20.66	0.002^**^	0.43
X_4_	58.38 ± 23.33	61.5 ± 24.68	0.354	−0.13
X_5_	67.07 ± 24.63	64.49 ± 26.68	0.472	0.1
X_6_	69.29 ± 26.85	63.55 ± 30.32	0.153	0.2
X_7_	64.85 ± 31.57	63.93 ± 33.13	0.838	0.03
X_8_	77.78 ± 28.41	72.9 ± 31.35	0.244	0.16
X_9_	60 ± 24.24	59.25 ± 23.3	0.822	0.03
X_10_	63.84 ± 22.98	60.75 ± 24.25	0.35	0.13
X_11_	61.82 ± 22.87	57.57 ± 24.83	0.204	0.18
X_12_	64.65 ± 25.04	71.59 ± 25.18	0.049^*^	−0.28
X_13_	64.44 ± 23.13	55.7 ± 28.02	0.016^**^	0.34
X_14_	63.84 ± 23.33	58.69 ± 26.39	0.141	0.21
X_15_	66.46 ± 21.73	59.07 ± 23.05	0.019^**^	0.33
X_16_	63.84 ± 25.34	58.13 ± 23.24	0.093	0.23
X_17_	65.86 ± 20.25	54.02 ± 25.36	0.000^***^	0.52
X_18_	63.64 ± 21.26	57.94 ± 23.78	0.072	0.25
X_19_	56.97 ± 24.3	64.49 ± 20.06	0.016^**^	−0.34
X_20_	74.58 ± 26.29	77.37 ± 23.15	0.421	0.11
**Each dimension score (weighting)**				
Balanced ability	3.13 ± 0.77	3 ± 0.64	0.187	0.18
Rhythm ability	11.96 ± 3.77	11.33 ± 4.17	0.258	0.16
Spatial orientation ability	6.82 ± 1.57	6.64 ± 1.51	0.401	0.12
Limb range of motion	3.23 ± 0.85	2.89 ± 0.89	0.005^**^	0.39
Body coordination ability	31.58 ± 8.11	27.69 ± 8.37	0.001^**^	0.47
Perceived judgment	8.43 ± 2.41	8.89 ± 2.3	0.161	−0.2
Total points	65.14 ± 10.83	60.43 ± 10.9	0.002^***^	0.43

X_1_, walking on tiptoes in a straight line; X_2_, walking on heels; X_3_, single-leg stance; X_4_, marching in place with eyes closed; X_5_, rhythmic sensitivity; X6, sequential reproduction; X_7_, fast/slow reproduction; X_8_, strong/weak reproduction; X_9_, curve walking; X_10_, repeated side sliding steps; X_11_, turning and changing direction in place; X_12_, directional kicking; X_13_, Single-leg seated forward bend (left); X_14_, single-leg seated forward bend (right); X_15_, shoulder stretch and touch (left); X_16_, shoulder stretch and touch (right); X_17_, jump square; X_18_, thrust fist and kick leg; X_19_, hand reaction time; X_20_, foot reaction time; ^*^P < 0.05, ^**^P < 0.01, ^***^P < 0.001.

**Table 17:** Gender differences in the development of children's coordination ability (5-year-old) ($\bar{X}\pm S$).

**Group**	**Female**	**Male**	***P*-value**	***d***
**Single metric score (unweighted)**				
X_1_	56.99 ± 24.38	60.00 ± 21.71	0.31	−0.13
X_2_	58.23 ± 25.15	61.54 ± 23.44	0.29	−0.14
X_3_	62.3 ± 22.32	57.08 ± 25.29	0.091	0.22
X_4_	57.54 ± 25.03	60.53 ± 22.59	0.332	0.13
X_5_	69.56 ± 21.73	67.23 ± 22.13	0.411	0.11
X_6_	78.23 ± 25.85	71.38 ± 26.11	0.042^*^	0.26
X_7_	75.04 ± 27.97	69.85 ± 28.64	0.155	0.18
X_8_	83.19 ± 26.13	83.08 ± 23.29	0.973	0.004
X_9_	57.35 ± 23.38	60.92 ± 24.00	0.242	−0.15
X_10_	60.88 ± 21.11	65.38 ± 25.97	0.143	−0.19
X_11_	59.47 ± 24.56	60.92 ± 22.81	0.633	−0.06
X_12_	44.96 ± 21.8	48.62 ± 23.88	0.216	−0.16
X_13_	69.03 ± 20.53	54.62 ± 22.24	0.000^***^	0.67
X_14_	69.38 ± 22.53	55.08 ± 21.14	0.000^***^	0.65
X_20_	62.65 ± 22.44	58.31 ± 24.4	0.152	0.19
X_16_	63.19 ± 22.92	59.38 ± 24.26	0.213	0.16
X_17_	60.53 ± 22.91	59.54 ± 25.08	0.749	0.04
X_18_	63.89 ± 22.18	58.92 ± 23.14	0.09	0.22
X_19_	56.81 ± 24.43	62.46 ± 24.02	0.071	−0.23
X_20_	41.95 ± 20.87	48.46 ± 24.13	0.026	−0.29
**Each dimension score (weighting)**				
Balanced ability	2.96 ± 0.70	2.96 ± 0.71	0.978	0
Rhythm ability	13.03 ± 3.61	12.46 ± 3.44	0.209	0.16
Spatial orientation ability	6.23 ± 1.37	6.59 ± 1.54	0.057	−0.25
Limb range of motion	3.27 ± 0.78	2.76 ± 0.85	0.000^***^	0.63
Body coordination ability	30.74 ± 8.79	28.98 ± 9.53	0.138	0.19
Perceived judgment	6.64 ± 2.54	7.42 ± 2.78	0.024^*^	−0.29
Total points	62.87 ± 11.45	61.17 ± 12.41	0.271	0.14

X1, walking on tiptoes in a straight line; X2, walking on heels; X3, single-leg stance; X4, marching in place with eyes closed; X5, rhythmic sensitivity; X6, sequential reproduction; X7, fast/slow reproduction; X8, strong/weak reproduction; X9, curve walking; X10, repeated side sliding steps; X11, turning and changing direction in place; X12, directional kicking; X13, single-leg seated forward bend (left); X14, single-leg seated forward bend (right); X15, shoulder stretch and touch (left); X16, shoulder stretch and touch (right); X17, jump square; X18, thrust fist and kick leg; X19, hand reaction time; X20, foot reaction time; ^*^P < 0.05, ^***^P < 0.001.

A detailed analysis of gender differences in the indicators for each component element reveals that, among the four balance ability indicators, boys scored higher than girls in X_4_ (marching in place with eyes closed), although the difference was not significant. Girls outscored boys in the other three indicators, with a significant difference observed only in X_3_ (single-leg stance; *P* < 0.01), which corresponds to a small-to-medium effect size (*d* = 0.43). This suggests that girls exhibit superior static balance compared to boys, while dynamic balance appears to be more evenly developed between the genders. Despite the higher mean score for girls, no significant gender disparity exists.

For all four rhythmic ability indicators, girls achieved higher scores than boys. However, the *t*-test indicated that there were no significant differences between genders in these indicators (refer to Table 16). Therefore, in 4-year-old children, there is no significant gender difference in rhythm perception and reproduction.

In the spatial orientation component of ability, boys achieved higher scores than girls in the directional kicking task (Test X_12_), with a significant difference (*P* < 0.05) and a small effect size (*d* = 0.28). This discrepancy may be attributed to boys greater inclination toward ball-related and control skill activities, and their regular engagement in sports may indirectly enhance their performance, despite the small effect size observed. For the remaining three indicators—curve walking, repeated side sliding steps, and turning and changing direction in place—girls obtained higher scores than boys. However, *t*-tests revealed no significant gender differences in these areas.

In all indicators of the component elements of range of limb movement, girls achieved higher scores than boys, with statistically significant differences observed in the left and right legs (X _13_, X _14_) and the left arm (X _15_; *P* < 0.05). These differences correspond to moderate effect sizes (*d* = 0.34, *d* = 0.33), indicating that girls exhibit greater flexibility in their limbs compared to boys.

Limb coordination ability, which reflects the synergistic cooperation of children's limbs, plays a crucial role in their coordination ability. Among the assessed indicators, girls outperformed boys in two specific tests. Statistical analysis using the *t*-test revealed a significant gender difference in the Jump square task (*P* < 0.001), with a medium effect size (Cohen's *d* = 0.52). This suggests that girls exhibited superior control over their limb movements.

Perceptual judgment primarily measures the simple reaction capabilities of young children, which are mainly determined by the reaction times of eye-hand and eye-foot coordination. Boys outperformed girls in both hand reaction time and foot reaction time tests. Statistical analysis using the *t*-test indicated a significant difference in hand reaction time (*P* < 0.05, effect size *d* = 0.34). However, no significant gender difference was observed in foot reaction time.

#### 3.5.2 The development level of coordination ability in 5-year-old children

The comprehensive evaluation results of the coordination ability of the 5-year-old group revealed that girls achieved a higher total score than boys; however, this difference was not statistically significant (*P* = 0.271). When examining the component element indicators, boys demonstrated higher scores than girls in perceptual judgment ability (*P* < 0.05, *d* = 0.29) and spatial orientation ability, although the latter did not reach statistical significance (*P* > 0.05). For the other indicators, boys scores were lower than those of girls. Girls excelled in balance ability, rhythmic ability, and the range of limb movement, with a particularly significant difference observed in the range of limb movement (*P* < 0.001, *d* = 0.63). This suggests that girls exhibit greater limb flexibility compared to boys, whereas boys tend to have superior reaction abilities.

Gender differences were analyzed across various indicators of balance and rhythmic abilities. For the balance indicators, boys achieved higher scores than girls in X_1_ (heel-to-toe walk), X_2_ (walking on heels), and X_4_ (marching in place with eyes closed). Conversely, girls excelled in X_3_ (single-leg stance). Despite these variations, *t*-test analyses indicated that there were no statistically significant gender differences in these balance-related indicators (*P* > 0.05).

Regarding rhythmic abilities, girls outperformed boys across all tested indicators. A significant gender disparity was noted in X_6_ (rhythmic sequence reproduction), with a *P*-value below 0.05, indicating a statistically significant difference. This difference, however, was characterized by a small effect size (*d* = 0.26), suggesting that although girls demonstrated superior performance in rhythm perception and reproduction, the magnitude of the gender difference was not substantial.

In the assessment results of spatial orientation ability, boys achieved higher scores than girls across all four indicators. Following an independent samples *t*-test (Table 17), it was determined that there was no statistically significant difference (*P* > 0.05). Thus, while boys demonstrated a higher average spatial orientation ability, the gender disparity was not statistically significant.

For the 5-year-old group in the assessment of the range of limb movement, girls outperformed boys in all component indicators. Significant differences were observed in X_13_ (single-leg seated forward bend left) and X_14_ (single-leg seated forward bend right), with *P*-values less than 0.001, and corresponding medium effect sizes (*d* = 0.67 and *d* = 0.65). This suggests a developmental advantage in flexibility for girls.

In the senior class children's performance, girls exhibited higher mean scores in both limb coordination ability indicators compared to boys; however, this difference was not statistically significant (*P* > 0.05). There was no clear gender disparity in the development of limb coordination ability.

Regarding the two perceptual judgment ability indicators—X_19_ (hand reaction time) and X_20_ (foot reaction time)—boys achieved higher scores than girls. A *t*-test indicated a significant difference in X_20_ (foot reaction time; *P* < 0.05), with a small effect size (*d* = 0.29), suggesting that boys had a quicker foot reaction speed. However, no significant gender difference was observed in X_19_ (hand reaction time; *P* > 0.05).

## 4 Discussion

### 4.1 The rationality analysis of the structural elements of coordination ability in young children

The coordination ability of young children are progressively honed and refined based on their innate genetic predispositions and levels of maturation (18). Studies in psychology suggest that these coordination ability enhance in tandem with the children's cognitive growth and are indicative of their ability to select and retain valuable motor experiences. Consequently, these coordination ability are showcase and enhanced through the acquisition of fundamental motor abilities, including walking, running, jumping, and throwing.

The renowned American psychologist, William James, posited that coordination is evidenced in motor skills through the body's fluidity and the harmonious movement of its various parts, characterized by precision in speed, stability, and aiming (19). Chinese scholar Hu (20) proposed that children's motor coordination encompasses the controlled manipulation of spatial and temporal dimensions, rhythm, and the intensity of force during the synchronization of different body segments. These abilities are marked by harmonious, fluid, efficient, and effortless movement (21). Diem (22) posits that children's coordination ability comprise elements such as balance, reaction, and spatial orientation. Coordination is closely related to neural development. Developing children's motor coordination ability significantly promotes the maturation of neural pathways, which, in turn, enhances their language, intelligence, and emotional skills (23). Coordination ability, as a subset of coordination, expands the concept by focusing on foundational skills (14). This study, grounded in previous research on coordination and motor abilities, examines the basic motor skills of young children. It suggests that coordinated movements are influenced by perceptual judgment, the range of limb movement, and limb coordination ability. Considering reaction ability as a component of perceptual judgment, the study concentrates on the kinematic characteristics and the factors that affect coordination ability in young children. Drawing on the theory of complex adaptive systems, we have constructed a framework for the coordination ability in young children. This framework encompasses dimensions such as balance ability, rhythmic ability, spatial orientation ability, perceptual judgment ability, and limb coordination ability, including the scope and precision of limb movements.

Balance ability is an important component of coordination ability in young children and is also a vital function for maintaining normal human activities. It is the ability to maintain the body in the required position during task execution and to keep body posture under control in various environments. For young children to move effectively and perform coordinated actions in different environments and tasks, they need the ability to maintain controlled body posture during both static and dynamic activities (24). Balance ability can be maintained by regulating the body's center of gravity to keep it within the boundaries of the support surface, thereby ensuring relative stability of posture. Moreover, balance ability influences the coordination of gross and fine motor actions such as sitting, standing, and walking (25, 26).

Good rhythmic ability enhances the coordination of various organ systems, improves the efficient contraction and relaxation of muscles, and makes movements more energy-efficient and effective. Young children who possess a strong sense of rhythm can accurately perceive and grasp the characteristics of rhythm, such as its duration, sequence, and intensity. In movement, they can perform actions more effectively, acquire skills rapidly, and exhibit enhanced coordination, making rhythmic ability an important component of their coordination ability. Spatial orientation ability significantly contributes to mastering movements and forming skills. Coordination disorders in children often stem from conflicts in temporal, force, and spatial organization of movements (27). The ability to perceive the spatial position of objects is already formed in early childhood. As an essential skill for adapting to their surroundings, young children's spatial orientation is vital for accurately recognizing their movement environment, determining their location and position, and swiftly adapting to perform various actions. This capability plays a crucial role in the development of responsive flexibility and coordination in movement (28).

The perceptual judgment ability of young children reflects the coordination and flexibility of their nervous and muscular systems during the execution of movements. This ability is crucial in responding to motor skills and motor potential (29). The perception of movement and information processing are closely related in the movement response process, playing a fundamental role in the learning and application of movement techniques. Moreover, there is a significant relationship between the speed of response and the accuracy of movement (30). Therefore, this ability is also a prerequisite for mastering coordination ability (31).

The simple movement patterns and early motor experiences during early childhood lay the foundation for the development of basic motor skills and coordination (11). Young children's coordination is evident in the harmony between their limbs, achieved through the synchronized cooperation of various nerves, systems, and organs. Limb coordination serves as an external trait and a vital aspect of a child's overall coordination. Consequently, Body coordination ability is a key element in assessing the coordination ability of young children.

Limb range of motion refers to the degree to which joints, ligaments, and soft tissues in the limbs can extend. The basic motor skills of young children primarily involve the execution of gross motor movements, with the flexibility of these movements largely determined by the range of motion in major joints such as the hips, knees, and shoulders. This flexibility is externally expressed through the range of limb movement. It influences the amplitude and aesthetic quality of limb movements during physical activities and plays a crucial role in minimizing the risk of injury. A lack of flexibility can diminish the quality of movement experiences and hinder the development of basic motor skills, potentially limiting the growth of essential coordination ability (32).

Drawing on the theory of Complex Adaptive Systems (CAS), we can consider coordination ability as a dynamic system. As this system engages in movement, its components actively adapt to various external information, including the environment, tasks, and individual circumstances. By engaging in continuous communication and adjustment, the system establishes an internal coordination mechanism, thus achieving a state of relative balance. Through this iterative process of communication and adaptation, the self-organizing behaviors of the systems components interact with the environment, resulting in optimized system performance and more harmonious movements.

### 4.2 Analysis of children's coordination ability development characteristics

(1) In the comprehensive evaluation of coordination ability, girls outperform boys, and gender differences are observed among 4-year-old (middle-class) children

The comprehensive scores for coordination ability across all age groups of young children indicated a trend where girls generally outperformed boys. Specifically, the gender disparity in the 4-year-old group was statistically significant, whereas no significant difference was found among the 5-year-old group. On the whole, girls demonstrated superior coordination ability compared to boys. In particular, the range of limb movement for girls across all age groups was significantly greater than that for boys, suggesting that girls possess better flexibility. This finding aligns with previous research, which has demonstrated that girls exhibit better flexibility than boys between the ages of 3 and 6 (33). This superior flexibility can be attributed to gender-based differences in muscle and ligament development, as well as to girls increased participation in dance activities. In terms of limb coordination ability, girls in the 4-year-old group significantly outperformed boys. In the 5-year-old group, although the average score of girls was higher than that of boys, the difference was not statistically significant. This suggests that girls limb coordination develops earlier than boys, but as they age, boys development tends to catch up. By the late preschool period, while girls still exhibited better scores on average than boys, the gender difference had become insignificant. Zhang (9) suggested that the period from ages 5 to 6 is a phase of rapid development for the coordination of upper and lower limbs in young children. This finding once again confirms that boys tend to experience faster growth in this area. Regarding perceptual judgment ability, boys consistently outperformed girls across all age groups, with particularly significant differences observed in the 5-year-old group. This highlights the gender disparities in hand-eye coordination, hand-foot coordination, and the flexibility of reactions during the early childhood stage. In the spatial orientation ability, boys also exhibited better performance than girls, although the differences were not statistically significant. This ability is associated with children's capacity to adapt to their environment, which involves making comprehensive judgments about the environment and ones position within it, as well as making timely adjustments to movements. Boys demonstrated a slight edge over girls in this aspect. In terms of balance and rhythmic abilities, girls outperformed boys. Previous studies have demonstrated that the sensitive period for the development of stability skills occurs at 5 years of age in boys and between 4 and 7 years of age in girls. Furthermore, girls tend to exhibit superior balance and rhythmic skills to boys during early childhood (10). Additional research has also suggested that, from the ages of 7 to 12, girls continue to demonstrate better balance and rhythmic abilities than boys, thereby lending support to the findings of the current study (34).

Coordination ability in early childhood develops rapidly, closely related to both innate physiological maturation and acquired motor experience. The developmental processes of boys and girls differ, resulting in an imbalance in the development of the components of coordination ability. Generally, girls tend to outperform boys in this area. However, research indicates that significant gender differences in basic motor skills become evident after the age of 8, with boys gradually surpassing girls (35).

(2) The development of the components of coordination ability exhibits gender asynchrony, with some indicators showing gender differences.

The overall level of coordination ability in young children is more developed in girls than in boys. Nonetheless, there are gender disparities in the progression of individual skills across different age groups. Girls tend to excel in areas such as balance, rhythmic abilities, the range of limb movements, and limb coordination. Conversely, boys tend to demonstrate greater proficiency in perceptual response and spatial orientation, particularly for those aged 5.0–5.9 years in senior kindergarten. With respect to balance skills, the single-leg standing test for children in middle and senior kindergarten indicates that girls outperform boys, with a notable gender disparity in the middle kindergarten class. This suggests that girls between the ages of 4.0 and 5.9 years have a superior static balance ability compared to boys. The activity of simultaneous marching with eyes closed illustrates that boys within the same age range generally exhibit superior dynamic balancing abilities compared to girls. Furthermore, as age progresses, there is a noticeable trend where boys begin to outperform girls in various indicators of dynamic balance, a finding that concurs with prior research (4, 36). This trend is further supported by studies that have highlighted significant gender disparities in the single-leg standing balance test among girls aged 4–8 years (8). The results suggest that girls tend to outperform boys in terms of rhythm ability, the range of limb movement, and limb coordination, aligning with earlier research findings. This superiority is attributed to the physiological traits of girls and is closely tied to their accumulated motor experiences. These attributes are frequently reflected in the dance movements that girls commonly engage in, representing one of the possible reasons for their advantage over boys. Conversely, boys exhibit stronger perceptual judgment, which translates into enhanced reaction times and better hand-eye and hand-foot coordination. This discrepancy is linked to both physiological and psychological differences, as well as to the pace at which they master actions and their receptivity to learning in the context of the test. Additionally, boys tend to perform better than girls on spatial orientation tasks, such as navigating curves, executing repeated side-stepping maneuvers, turning on the spot while walking, and performing directional kicks. The data indicates that boys tend to have an advantage in distinguishing directions, positioning themselves, and changing directions swiftly, suggesting enhanced stability of their vestibular organs. Additionally, the ability to kick in a specific direction is an operational skill. Research demonstrates that the sensitive period for developing such skills begins after the age of 4, and during this period, girls typically develop these skills at a slower pace than boys. Furthermore, the observed gender disparities in the research findings can also be attributed to boys greater experience with ball games, which provides them with more opportunities to hone their motor skills (37).

The development of coordination ability in young children is comprehensive. It is crucial to pay attention to the levels of each constituent element, ensuring they interact and integrate to enhance overall coordination ability. In children aged 4–6, girls generally exhibit higher levels of coordination than boys, with gender disparities noticeable in each skill component. It is essential to acknowledge both the commonalities in children's developmental trajectories and the variations in their pace of development. This understanding should inform tailored guidance to help each child progress from their current level. In children's activities, it is essential to fully understand the comprehensive development of coordination ability and recognize the differences between genders. Considering the unique aspects of motor development in early childhood, we must create favorable conditions, be attentive to individual differences, and employ diverse methods to ensure that each child's coordination ability develop suitably.

## 5 Conclusion

The evaluation tool developed is designed to accurately and comprehensively assess the developmental level of coordination ability in young children. Girls generally demonstrate better performance in the overall assessment of coordination ability compared to boys, with notable gender differences particularly among 4-year-olds. The development of the various components of coordination ability shows asynchrony between genders, with some measures displaying disparities. By analyzing individual progress over time (longitudinal performance) in comparison to the performance of the entire class (horizontal performance), we can establish a framework for evaluating each child's actual level of coordination ability. This method enables the timely detection of any shortcomings and allows for the adoption of more targeted training strategies.

## 6 Limitations and future directions

(1) Although this study developed an assessment tool for coordination ability in children aged 4.0–5.9 years and revealed their developmental characteristics, several limitations should be noted. First, measurement invariance tests (including configural, metric, and scalar invariance) across sex and age groups were not conducted. This prevents us from fully ensuring the consistency of measurement scales of indicators across different groups, which may introduce slight biases into the interpretation of gender differences and age-related developmental differences. Second, due to limitations in the original data collection design, after adopting stratified random cluster sampling, cluster identifiers such as kindergarten codes were not fully recorded. This made it impossible to calculate Intraclass Correlation Coefficients (ICC) to quantify clustering effects, nor to address data non-independence through methods such as cluster-robust standard errors or design effect correction, potentially leading to slight biases in the statistical inference of group comparisons. Third, data collection and core analyses were conducted several years ago, and the original protocol did not measure potential confounding variables such as socioeconomic status (SES), prior motor training experience, or differences in preschool curricula. Additionally, the original data are no longer accessible, precluding *post-hoc* statistical adjustments for these factors, and some observed group differences may be influenced by uncontrolled variables. Furthermore, the sample did not adequately represent populations from diverse SES and educational backgrounds, which may limit the generalizability of the findings. Future studies should: ① conduct measurement invariance tests to verify the consistency of indicators across different gender and age groups; ② standardize the recording of cluster identifiers and adopt rigorous statistical methods such as mixed-effects models to address data non-independence; ③ incorporate confounding variables and improve sample representativeness through stratified sampling.
(2) The current validation approach for the evaluation criteria has limitations. This study only conducted back-testing validation through a single data split (80% modeling set, 20% testing set). Although the overall misclassification rate was below 5%, the stability and generalizability of this error rate warrant further investigation. Future work should employ more robust techniques such as *k*-fold cross-validation to reduce model overfitting to specific data partitions and enhance the validity and generalizability of the evaluation criteria.

## Data Availability

The original contributions presented in the study are included in the article/Supplementary material, further inquiries can be directed to the corresponding author.
