# Supplementary material for: Construction and application of coordination ability evaluation tool for children aged 4–5.9 years old
Source: Front Public Health. 2026 Feb 11;14:1698428. doi: 10.3389/fpubh.2026.1698428 (PMC12932535; doi:10.3389/fpubh.2026.1698428)
Supplement: Supplementary file 1 [file Table_1.pdf]

Appendix

Table 1 Test-Retest Reliability （ $\bar{X} \pm SD$，n=34）

Test Indicator	Pretest	Posttest	r	Test Indicator	Pretest	Posttest	r
Walking on tiptoes in a straight line（s）	5.02±1.34	5.08±2.33	0.856	Turning and Changing Direction in Place（s）	21.7±2.78	16.01±6.01	0.813
walking on heels（s）	5.65±1.71	5.73±2.35	0.898	Directional Kicking（time）	5.47±1.76	5.52±2.18	0.967
single-leg stance（s）	27.7±35.5	28.7±17.8	0.914	Single-Leg Seated Forward Bend (Left)（cm）	10.49±3.16	10.75±5.6	0.946
Marching in place with eyes closed（s）	20.9±14.7	18.72±12.98	0.836	Single-Leg Seated Forward Bend (Right)（cm）	11.2±3.56	10.02±5.57	0.897
Rhythmic Sensitivity（minute）	3.17±1.6	4.38±1.14	0.827	Shoulder Stretch and Touch (Left)（cm）	20.9±3.64	16.84±5.68	0.812
Sequential Reproduction（minute）	3.08±1.5	3.54±1.38	0.916	Shoulder Stretch and Touch (Right)（cm）	19.1±4.74	16.89±5.57	0.878
Fast/Slow Reproduction（minute）	2.97±1.67	3.43±1.52	0.874	Jump square（s）	10.61±3.58	10.1±3.49	0.902
Strong/Weak Reproduction（minute）	3.41±1.79	3.98±1.37	0.867	thrust fist and kick leg（time）	22.8±12.89	23.1±14.2	0.923
Curve Walking（s）	5.85±0.68	5.71±1.20	0.935	hand reaction time（cm）	29.5±3.89	30.6±5.98	0.945
Repeated Side Sliding Steps（time）	30±3.21	31.35±4.26	0.956	foot reaction time（cm）	35.8±4.26	34.3±5.57	0.958

Table 2 Effect of age on indicators

Dependent Variable	III-Sum of Squares	mean square	F	P	Bias Eta Square
x_1	0.429	0.429	4.249	0.021	0.128
x_2	26.150	26.150	11.706	0.002	0.274
x_3	2064.400	2064.400	1.621	0.212	0.050
x_4	4.809	4.809	0.021	0.886	0.001
x_5	5.236	5.236	4.302	0.046	0.122
x_6	8.365	8.365	3.936	0.040	0.113
x_7	29.192	29.192	15.139	0.000	0.328
x_8	36.465	36.465	17.724	0.000	0.364
x_9	0.186	0.186	4.373	0.046	0.122
x_{10}	50.683	50.683	5.429	0.026	0.149
x_{11}	33.643	33.643	4.894	0.034	0.136
x_{12}	1.383	1.383	4.434	0.015	0.114
x_{13}	0.033	0.033	0.003	0.956	0.000
x_{14}	3.386	3.386	0.253	0.618	0.008
x_{15}	60.879	60.879	5.071	0.032	0.141

x_{16}	185.467	185.467	10.340	0.003	0.250
x_{17}	90.431	90.431	8.492	0.007	0.215
x_{18}	1585.868	1585.868	12.816	0.001	0.292
x_{19}	19.069	19.069	1.248	0.273	0.039
x_{20}	21.347	21.347	1.342	0.254	0.043

Note:X1=Walking on tiptoes in a straight line, X2=walking on heels, X3=single-leg stance, X4=Marching in place with eyes closed, X5= Rhythmic Sensitivity, X6= Sequential Reproduction, X7= Fast/Slow Reproduction, X8= Strong/Weak Reproduction, X9= Curve Walking, X10= Repeated Side Sliding Steps, X11= Turning and Changing Direction in Place, X12= Directional Kicking, X13= Single-Leg Seated Forward Bend (Left), X14= Single-Leg Seated Forward Bend (Right), X15= Shoulder Stretch and Touch (Left), X16= Shoulder Stretch and Touch (Right), X17=Jump square; X18=thrust fist and kick leg; X19=hand reaction time; X20=foot reaction time

Table 3 Core Dimensions and Testing Methods of Coordination Ability

Core Dimension	Test Indicators	Measurement Method
Balance Ability	1. Tiptoe straight-line walking<br>2. walking on heels<br>3. Single-leg stance<br>4. Marching in place with eyes closed	1. Time to complete 5m tiptoe walk<br>2. Time to complete 5m heel walk<br>3. Duration of single-leg standing (max 60s)<br>4. Duration of eyes-closed marching (max 60s)
Rhythm Ability	1. Rhythmic Sensitivity<br>2. Sequential Reproduction<br>3. Fast/Slow Reproduction<br>4. Strong/Weak Reproduction	1. Score (1–5) for perceiving music rhythm<br>2. Score (1–5) for reproducing given rhythm sequences<br>3. Score (1–5) for adjusting movement speed to match rhythm<br>4. Score (1–5) for adjusting movement intensity to match rhythm
Spatial Orientation Ability	1. Curve walking<br>2. Repeated side sliding steps<br>3. In-place turning and direction change<br>4. Directional kicking	1. Time to complete 5m curved path walk<br>2. Number of side slides in 30s<br>3. Time to complete 3 in-place turns<br>4. Number of accurate kicks to a target (3m away)
Perceptual Judgment Ability	1. Hand reaction time<br>2. Foot reaction time	1. Distance of hand movement to catch a falling ruler (shorter = faster reaction)<br>2. Distance of foot movement to step on a light sensor (shorter = faster reaction)
Limb Coordination Ability	1. Jump square<br>2. Thrust fist and kick leg	1. Time to jump through a 3×3 grid (5 rounds)<br>2. Number of synchronized fist-thrust and leg-kick movements in 30s
Limb Movement Range	1. Single-leg seated forward bend (left/right)<br>2. Shoulder stretch and touch (left/right)	1. Distance (cm) of finger reach beyond toe tips (seated, single-leg extended)<br>2. Distance (cm) between hands when stretching across the back (shoulder height)

Table 4 Inter-Rater Reliability of Coordination Ability

Level 1 Dimension	Individual Indicator	ICC(2,1)	95% Confidence Interval
Balance Ability	Tiptoe Straight-LineWalking (X1)	0.923	[0.876, 0.954]
	Heel Straight-Line Walking (X2)	0.917	[0.868, 0.949]
	Single-Leg Stance (X3)	0.896	[0.839, 0.935]
	Eyes-Closed Marching in Place (X4)	0.882	[0.819, 0.927]
Rhythm Ability	Rhythmic Sensitivity (X5)	0.875	[0.809, 0.922]
	Sequential Reproduction (X6)	0.903	[0.851, 0.939]
	Fast/Slow Reproduction (X7)	0.864	[0.795, 0.916]
	Strong/Weak Reproduction (X8)	0.887	[0.826, 0.929]
Spatial Orientation	Ability Curve Walking (X9)	0.931	[0.889, 0.958]
	Repeated Side Sliding Steps(X10)	0.912	[0.861, 0.946]
	Turning and Changing Direction in Place (X11)	0.898	[0.842, 0.936]
	Directional Kicking (X12)	0.945	[0.908, 0.966]
Limb Movement Range	Single-Leg Seated Forward Bend (Left, X13)	0.928	[0.883, 0.956]
	Single-Leg Seated Forward Bend (Right, X14)	0.935	[0.894, 0.960]
	Shoulder Stretch and Touch (Left, X15)	0.879	[0.815, 0.924]
	Shoulder Stretch and Touch (Right, X16)	0.885	[0.823, 0.928]
Limb Coordination Ability	Jump Square (X17)	0.907	[0.857, 0.941]
	Thrust Fist and Kick Leg (X18)	0.915	[0.865, 0.948]
Perceptual Judgment Ability	Hand Reaction Time (X19)	0.942	[0.905, 0.965]
	Foot Reaction Time (X20)	0.938	[0.899, 0.962]
Composite Dimension	——	0.926	
